# Biostimulant potential of triacontanol extracted from a legume-derived hydrolysate in *Eruca sativa* (L.)

**DOI:** 10.3389/fpls.2026.1847175

**Published:** 2026-06-30

**Authors:** Michela Schiavon, Paola Ganugi, Veronica Zuffi, Begoña Miras-Moreno, Serenella Nardi, Andrea Ertani

**Affiliations:** 1 Dipartimento di Scienze Agrarie, Forestali e Alimentari (DISAFA), Università di Torino, Grugliasco, Torino, Italy; 2 Dipartimento di Scienze e Tecnologie Agro-Alimentari, Alma Mater Studiorum, University of Bologna, Bologna, Italy; 3 Department of Plant Biology, Faculty of Biology, University of Murcia, Murcia, Spain; 4 Dipartimento di Agronomia, Animali, Alimenti, Risorse Naturali e Ambiente (DAFNAE), Università di Padova, Legnaro, Padova, Italy

**Keywords:** FT-IR spectroscopy, *Medicago sativa* L., metabolomics, rocket, supercritical CO_2_

## Abstract

Triacontanol (TRIA) is a long-chain aliphatic alcohol naturally occurring in plant epicuticular waxes and used as a biostimulant in agriculture to enhance plant health and yield. However, its effectiveness may vary depending on the source and extraction method. Thus, in this study, three sources of triacontanol were tested: a synthetic product (TRIA0), and two extracts (TRIA1 and TRIA2) obtained from a commercial legume-derived hydrolysate using supercritical CO_2_ extraction at different conditions. Following chemical and spectroscopic characterization, the three TRIA samples were tested for their biostimulant effects on hydroponically grown rocket (*Eruca sativa* L.) plants under controlled conditions. A standardized concentration of TRIA (2 mg L^-1^) was applied to assess the effects of the source matrix. The results demonstrated that extraction conditions strongly influenced TRIA efficacy. In particular, TRIA1 was the most effective in increasing the leaf and root fresh weight, SPAD values, nutrient accumulation (especially Ca, Fe, P, and S), and total phenols. By contrast, the higher extraction temperature used for TRIA2 appeared to partially compromise its activity, resulting in weaker physiological and metabolic responses, lower phenolic accumulation, and reduced stimulation of metabolic pathways. Metabolomic analysis evidenced treatment-dependent differences in metabolite profiles, particularly in pathways related to fatty acid, secondary metabolism, stress tolerance and growth regulation. These findings demonstrate the value of metabolomics in elucidating the molecular mechanisms underlying biostimulant action and in identifying key pathways activated by TRIA treatments and highlight the importance of extraction methods in determining TRIA performance and the potential of optimized legume-derived TRIA formulations to enhance crop responses linked to productivity.

## 1 Introduction

Triacontanol (TRIA) is a long-chain aliphatic alcohol that in the last years has garnered significant attention as a biostimulant for its remarkable growth-promoting properties when applied exogenously to plants (Naeem et al., 2011; Colautti et al., 2023; Parrey et al., 2023). In Asia, it is primarily used to enhance rice production, but its application is rapidly expanding in Western countries (Manai et al., 2024).

Naturally occurring in epicuticular waxes and beeswax, TRIA has been widely reported to stimulate plant growth by enhancing key physiological and biochemical processes, including photosynthesis, carbon and nitrogen metabolism, and water and nutrient uptake (Chen et al., 2003; Naeem et al., 2011; Ertani et al., 2013; Nanda et al., 2022; Parrey et al., 2023). TRIA improves photosynthetic performance by increasing PSII efficiency, Rubisco activity, and chlorophyll content (Sarwar et al., 2021; El-Beltagi et al., 2022). It also modulates phytohormone levels and enhances the accumulation of amino acids, soluble sugars, and proteins (Verma et al., 2022). Furthermore, TRIA has been shown to improve plant tolerance to abiotic stresses, such as salinity, drought, heavy metals, and extreme temperature, by stimulating antioxidant enzyme activity and secondary metabolite production, thereby supporting cellular integrity and function (Ertani et al., 2013; Sarwar et al., 2021; Weremczuk-Jeżyna et al., 2022). Despite these well-known functions in plants, the molecular mechanisms that explain TRIA mode of action and its role in plant signaling and perception is still partly unclear. It has been speculated that TRIA induces the synthesis of 9-L-adenosine, which activates the cytoplasmic Ca^2+^-dependent phosphorylation signaling pathway regulating transcription factors such as MYB2, CAMTA3, GTL, while promoting the expression of photosynthesis-related and other associated genes (Ries et al., 1993; Verma et al., 2022; Parrey et al., 2023). Foliar application of TRIA, in particular, is known to elevate L(+)-adenosine levels, which triggers various physiological responses, including enhanced malate dehydrogenase activity and overall plant growth (Islam and Mohammad, 2020).

TRIA activity in plants can vary based on the extraction method and source matrix (e.g., legume-derived hydrolysates, epicuticular waxes). Extraction techniques could affect TRIA purity by altering the content of secondary compounds, such as flavonoids or fatty acids, which may synergistically enhance or modulate its effectiveness. Supercritical CO_2_ extraction tends to produce a relatively high purity compound, while other methods may leave more solvent residues or co-extract bioactive metabolites. Additionally, some procedures may alter TRIA structure, reducing its solubility and bioavailability, as well as its biostimulants efficiency (Ali et al., 2021).

Rocket *(Eruca sativa* L.) is a widely consumed leafy vegetable (Yang et al., 2021), valued for its high nutritional quality and abundance of functional compounds including glucosinolates and flavonoids which are associated with multiple health benefits (Buitrago-Villanueva et al., 2023; Schiavon et al., 2022; Lee et al., 2024). Its adaptability to diverse cultivation systems, including hydroponics, supports its importance in modern sustainable agriculture (Drygaś et al., 2024).

Recent studies have demonstrated that the nutritional quality of rocket can be further enhanced through sustainable cultivation practices involving the use of biostimulants (Giordano, 2021; Mollo et al., 2025). In particular, the application of *Ascophyllum nodosum* extracts have been reported to determine to significantly enhance the content of several health-promoting metabolites like total phenolics and flavonoids, and in antioxidant activity in rocket microgreens, thereby increasing their potential benefits for human health (Drygaś et al., 2024).

The analysis of metabolic changes in horticultural crops following biostimulant application is relevant for understanding product efficacy and formulation performance (Alseekh and Fernie, 2018). Metabolomics enables the comprehensive characterization of plant metabolic responses to environmental factors and treatments, providing insights into biochemical pathways, stress responses, and potential biomarkers (Fiehn, 2002). In this framework, several studies have demonstrated that biostimulants, such as seaweed extracts, protein hydrolysates, and microbial formulations, can significantly modify plant metabolic profiles, enhancing the accumulation of compounds involved in stress tolerance and growth regulation (Shukla et al., 2019; Ertani et al., 2013; Lucini et al., 2015). More recently, metabolomic analyses have shown that TRIA treatments can alter pathways related to lipid metabolism, secondary metabolite biosynthesis, and hormone regulation, with responses strongly influenced by formulation and extraction methods (Islam and Mohammad, 2020; Manai et al., 2024).

Therefore, the aim of this study was to evaluate the biostimulant effects of TRIA of different origin and produced under varying extraction procedures and conditions in rocket plants. In doing so, the study shows that the biostimulant effects of TRIA depend not only on the active compound itself but also on the extraction method and source matrix. By linking processing conditions to plant metabolic responses, the study highlights that TRIA-based products are not functionally equivalent, and that formulation plays a key role in determining their efficacy.

## 2 Materials and methods

### 2.1 Characterization of the products via FT-IR and elemental analysis

This study evaluated three sources of triacontanol: TRIA0, a synthetic product, and TRIA1 and TRIA2, which were extracted from a commercial legume-derived hydrolysates using supercritical CO_2_ extraction under different extraction conditions and subsequently solubilized and re-suspended. The legume source was *Medicago sativa* L. TRIA1 was extracted maintaining the temperature in the range 40-60°C, while TRIA2 was extracted between 60 and 80°C. The pressure during the extraction procedure was maintained within 200–300 bar for both TRIAs. Before analysis, TRIA was dissolved in warm ethanol and then diluted into the treatment solution, resulting in a final ethanol concentration of 0.1% (v/v). The three products were first analyzed spectroscopically via Fourier-transform infrared (FT-IR) spectroscopy and elemental analysis to assess their functional group and element composition. A more detailed characterization of the products cannot be disclosed due to the proprietary nature of the extraction process and composition of these materials.

FT-IR spectroscopy was performed using a Bruker Tensor FT-IR instrument (Bruker Optics, Ettlingen, Germany) equipped with an accessory for analysis in micro-Attenuated Total Reflection (ATR). The sampling device contained a microdiamond crystal, a single reflection with an angle of incidence of 45° (Specac Quest ATR, Specac Ltd., Orpington, Kent, UK). Spectra were acquired between 4000 and 400 cm^-1^, with a spectral resolution of 4 cm^-1^ and 64 scans. Background spectra were taken against air under the same conditions before each sample. Spectra were processed with the Grams/386 spectroscopic software (version 6.00, Galactic Industries Corporation, Salem, NH, USA. The relative functional groups assigned in the spectra are reported in **Supplementary Table 1**.

The elemental characterization was performed on the lyophilized products. Total C and N contents were determined using a dry combustion procedure inside an element analyzer (vario MACRO CNS, Hanau, Germany). The pH was determined in water (3:50 w/v), as well as the electrical conductivity (EC) (1/10 w/v).

### 2.2 Plant material and growth conditions

Rocket plants were initially grown in half-strength MS agar medium (Murashige and Skoog, 1962) inside a climate-controlled chamber with a photoperiod of 14 h light/10 h dark, a temperature regime of 26/21 °C (day/night), relative humidity of 70/85%, and a photon flux density (PFD) of 280 µmol m^-^²s^-^¹. Once the plants reached approximately 6–8 cm in height, they were transferred in pots containing a perlite substrate for three weeks within a hydroponic setup. During the first week, the plants were grown in a 1:4 strength Hoagland solution for acclimation, then they were supplied with a half-strength Hoagland solution since the second week. The nutrient solution was renewed every 48 h and had the following composition (mM): 0.63 KH_2_PO_4_, 2 Ca(NO_3_)_2_, 3 KNO_3_, 1.5 MgSO_4_, 0.040 FeNaEDTA, plus micronutrients. Three-week-old plants were supplied with the TRIA products by addition to the nutrient solution. Each TRIA formulation was applied as a single treatment, which delivered an equivalent amount of triacontanol, corresponding to 2 mg L^-^¹. This dosage was previously established through preliminary tests conducted in plants to identify the optimal working concentration (data not shown). The pH and electrical conductivity (EC) of the nutrient solution was monitored and did not change after the addition of TRIA products. The experiment followed a completely randomized design including four treatments: control (TRIA untreated), TRIA0, TRIA1, TRIA2. Each treatment comprised five independent biological replicates (pots), with one plant per pot. The experiment lasted 30 days and was conducted in duplicate. In the first experiment, all five plants per treatment were used for biomass and element quantification. For fresh biomass estimation, plants of each treatment were carefully washed, dried with blotting paper, separated in leaves and roots and individually weighed (n = 5). Then, the plant material was dried in the oven at 60 °C for three days and the dry weight was recorded. In the second experiment, three representative plants per treatment were selected for biochemical and metabolomic analyses; these samples were immediately frozen in liquid nitrogen after sampling and stored at –80 °C until analyses.

### 2.3 SPAD index, total protein and phenol quantification

SPAD index determination was performed using the SPAD-502 Leaf Chlorophyll Meter (Minolta Camera Co., Ltd., Osaka, Japan) on the last expanded leaf of three rocket plants per treatment (n=3).

For the extraction of soluble proteins, 500 mg of frozen foliar and root tissues from three plants per treatment (n=3) were ground in liquid nitrogen to maintain protein integrity. The resulting powder was then vortexed with 5 mL of an extraction buffer containing 100 mM Tris-HCl (pH 7.5), 1 mM Na_2_EDTA, and 5 mM DTT, which helps stabilize the proteins and prevent oxidation. The homogenate was subjected to centrifugation at 14,000 g for 15 minutes at 4 °C to separate the soluble proteins from the cellular debris. The supernatant obtained was treated with 10% (w/v) trichloroacetic acid (TCA) to precipitate the proteins and then centrifuged again to collect the protein pellets. The pellets were washed with cold acetone to remove residual TCA and lipids and briefly air-dried. The dried protein pellets were re-suspended in 0.1 N NaOH to solubilize the proteins fully. The protein concentration was determined using the Bradford method (Bradford, 1976) by measuring the absorbance at 595 nm with a UV/VIS spectrophotometer (Lambda 1, Perkin-Elmer, Monza, Italy). The final protein content was expressed as mg of protein per gram of fresh weight, allowing for an accurate assessment of soluble protein levels in the tissues analyzed.

Soluble phenolic acids were extracted from three plants per treatment (n=3) by grinding 1 g of fresh leaf tissue in a mortar with pure methanol in a ratio of 1:3 (w/v). The homogenate was then placed in an ice bath for 30 minutes to minimize oxidation of the phenolic compounds. Following extraction, the samples were centrifuged at 5,000 g for 30 minutes at 4 °C to separate the phenolic-rich supernatant from the solid residue. The collected supernatants were stored at -20 °C until further analysis. Total phenolic content was quantified using the method of Arnaldos et al. (2001). Specifically, 1 mL of 2% Na_2_CO_3_ solution and 75 μL of Folin-Ciocalteau reagent (Sigma-Aldrich) were added to 100 μL of the phenolic extract. The mixture was incubated for 15 minutes at 25 °C in the dark to develop color. Absorbance was then measured at 725 nm using a spectrophotometer. Catechin was used as a standard for calibration, following the procedure described by Meenakshi et al. (2009), allowing the phenolic content to be expressed in terms of catechin equivalents.

### 2.4 Elemental analysis

Dried leaves and roots of five rocket plants per treatment were ground before elemental analysis. The digestions were carried out as described in Ertani et al. (2011), inside closed Teflon vessels of 120 mL volume using approximately 500 mg dry leaf or root material and 10 mL of 30% (v/v) HCl. After digestion, the resulting solution was transferred and diluted with 10 mL ultrapure water. The mineralized samples were then diluted with ultrapure water, and each element was analyzed by Inductively Coupled Plasma–Optical Emission Spectroscopy (ICP-OES) (Spectro Amatek Arcos II ICP-OES, Kleve, Germany). The analyses were performed in triplicate.

### 2.5 Metabolomic analysis

Frozen leaf material from three plants per treatment (n =3) was ground in liquid nitrogen and 1 gram of ground tissue was extracted in 10 mL of 0.1% formic acid in 80% methanol using an Ultra-Turrax homogenizer (Ika T-25, Staufen, Germany). The extracts were centrifuged at 4 °C for 10 min at 12,000 g, and the supernatants filtered on 0.22 µm cellulose syringe filters. Finally, 1 mL of each sample extract was transferred to vials and stored at -20 °C until further analysis. To ensure data reliability, procedure blanks (containing only the methanolic extraction solution) and quality control (QC) samples were regularly injected throughout the analytical sequence, specifically at the beginning, at the end of the batch, and after every ten samples. QC samples were generated by pooling 20 µL from each individual supernatant and served to evaluate both the robustness of the chemometric analyses and the consistency of the instrumental performance. Procedure blanks were incorporated into the statistical analysis to identify and eliminate background interferences. The screening of plant metabolites was conducted using a hybrid quadrupole-time-of-flight (Q-TOF) mass spectrometer coupled with an UHPLC system (UHPLC/Q-TOF) in positive scan mode, acquiring spectra in the 100–1600 m/z range. An Agilent 1290 liquid chromatograph, equipped with a binary pump and a Dual Electrospray JetStream ionization system, was coupled to an Agilent G6550 mass spectrometer. Reverse-phase separation was performed on an Agilent Zorbax Eclipse-plus column (75 × 2.1 mm, 1.8 μm). The mobile phases were water (A) and methanol (B), both LCMS grade, with 0.1% formic acid and 5 mM ammonium formate. The gradient increased from 5% to 90% B over 30 min, holding for 5 min. The mobile phase temperature was 35 °C, with an injection volume of 3 μL and a flow rate of 220 μL/min. Source conditions included a sheath gas flow of N at 10 L min^-^¹ (350 °C), drying gas at 10 L min^-^¹ (280 °C), a nebulizer pressure of 60 psig, a nozzle voltage of 300 V, and a capillary voltage of 3.5 kV. Lock masses (m/z 121.0509 and 922.0098) were continuously infused throughout the chromatographic run.

Q-TOF raw data were processed using MassHunter Qualitative Analysis B.05 (Agilent Technologies) with the “find-by-molecular-feature” algorithm. Compound identification was based on accurate mass and isotope pattern, expressed as an overall identification score. Molecular features underwent recursive analysis using Profinder B.05 (Agilent Technologies) for alignment and filtering after initial deconvolution. Features absent in at least 80% of replicates within one treatment were discarded. The combination of monoisotopic mass, isotope ratio, and their distribution allowed annotation of 122 compounds against the PlantCyc 12.6 reference database (Plant Metabolic Network, http://www.plantcyc.org). Metabolite annotation was performed at putative Level 2 confidence according to MSI guidelines, based on accurate mass, isotope pattern, and database matching, without confirmation using authentic standards (Sumner et al., 2007).

The peak volume of each compound, identified with a mass accuracy below 5 ppm and an overall identification score above 80/100, was extracted from the total ion current and exported for statistical analysis and interpretation.

### 2.6 Statistics

For growth, SPAD index, elemental composition, protein, and phenolic content, data were analyzed by one-way ANOVA using SPSS software version 26.0 (SPSS, Chicago, IL, USA). Data normality and homogeneity of variance were verified using the Shapiro–Wilk and Levene tests, respectively. When significant differences were detected, means were separated using Tukey’s HSD *post hoc* test for multiple comparisons at p < 0.05. All data are expressed as means ± standard error.

Metabolomic data were initially processed using Agilent Mass Profiler Professional B.12.06 (Agilent Technologies, Santa Clara, CA, USA) for normalization, baseline correction and preliminary data exploration. Unsupervised hierarchical clustering (HCA), based on fold-change heatmaps and Squared Euclidean distance, was performed to identify natural clustering patterns among treatments. Supervised multivariate analysis was subsequently carried out using SIMCA 13 (Umetrics, Malmö, Sweden), applying orthogonal projections to latent structures discriminant analysis (OPLS-DA). Model validity was confirmed using CV-ANOVA, with overfitting assessed via permutation testing (>100), while model performance was evaluated by R²Y (explained variance) and Q²Y (predictive ability). Metabolites contributing most to class separation were identified through variable importance in projection (VIP) scores derived from the OPLS-DA model, using a threshold of VIP ≥ 1.3. Independently, differential metabolites were identified through univariate analysis combining one-way ANOVA, Benjamini–Hochberg false discovery rate (FDR) correction, and fold-change filtering (FC > 1; adjusted p < 0.05). The subset of significantly modulated metabolites obtained from the univariate analysis was subsequently used for pathway mapping through the Omic Viewer Pathway Tool within the PlantCyc platform (Caspi et al., 2014).

## 3 Results

### 3.1 FT-IR spectroscopic and chemical characterization of the TRIA products

FT-IR spectroscopy was employed in this study as an analytical tool to verify the presence of characteristic functional groups and to establish consistent spectral fingerprints for each TRIA product. Although FT-IR alone does not provide a comprehensive assessment of purity, relative abundance, or minor oxidation products when present in very low amounts as expected by CO_2_ processing approach, it enables the identification of reproducible differences in functional group composition among samples.

**Figure 1:**
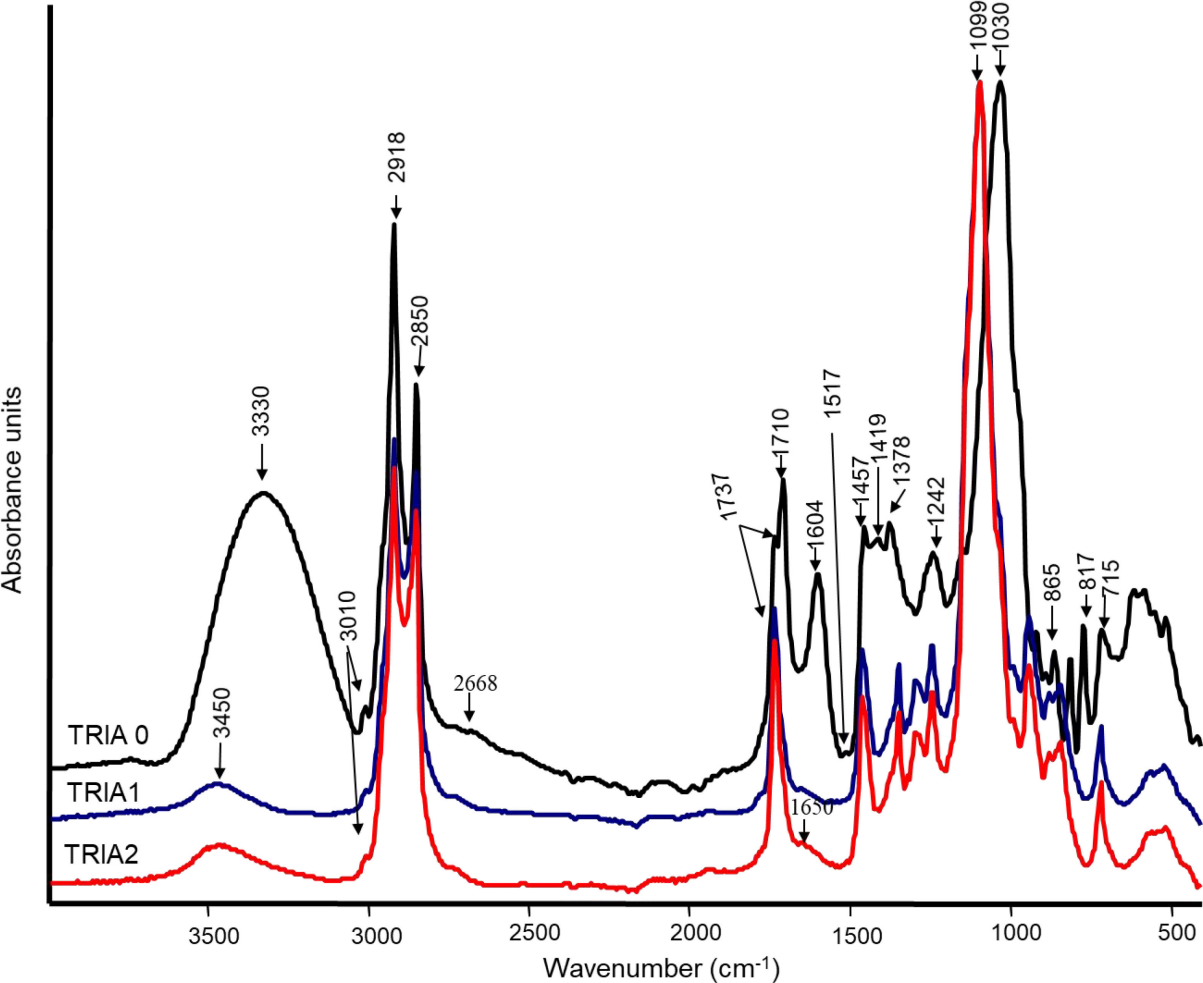
FT-IR spectra of TRIA0, TRIA1 and TRIA2.

As displayed in **Figure 1**, the FT-IR spectra of TRIA0, TRIA1 and TRIA2 exhibit characteristic bands associated with long-chain alcohols. In particular, the O-H stretching vibrations (3650–3200 cm^-1^) and the vibrations of the CH groups (2960–2852 cm^-1^) are of particular note. These CH absorptions correspond to the asymmetric (2960 cm^-^¹) and symmetric (2852 cm^-^¹) stretching modes of the methylene (CH_2_) groups along the alkyl chains. The bands are also coupled to CH_2_ bending vibrations at 1457 cm^-1^ and OH bending at 1378 cm^-1^. A weak shoulder at 2957 cm^-^¹ further suggests the presence of CH_3_ stretching vibrations from terminal methyl groups. The intense band observed between 1100 and 1000 cm^-^¹ is attributed to C–O stretching, coupled with other skeletal vibrational modes (Rao, 1963). This region is characteristic of the alcohol functional group and is correlated with chain length (Rao, 1963). Additionally, the band located near 720 cm^-1^ is attributed to CH_2_ rocking vibrations, which are typically observed when the number of methylene groups exceeds two (Rao, 1963).

All spectra also showed other bands not related to the structure of saturated free alcohol. For instance, absorptions at 3010 cm^-^¹ correspond to C=CH stretching vibrations, while the band at 1737 cm^-^¹ indicates R–C=O stretching in esters. Additional features in the 1650–1500 cm^-^¹ region arise from C=O stretching in acids as well as C=C vibrations in aliphatic chains and aromatic rings. The band at 1242 cm^-^¹ is likewise characteristic of C–O stretching in esters (Bellamy, 1975). As shown in **Figure 1**, the spectral profiles of TRIA 1 and TRIA 2 are found to be superimposable, but with very slight variations observable. In TRIA 2, the bands at 3450 cm^-1^ and the shoulder at 1650 cm^-1^ show greater broadening and a marginal increase in relative intensity. This could be indicative of an increased presence of hydroxyl and carbonyl groups in acids, as well as of -C=C- (cis-) bonding (Bellamy, 1975).

The spectrum of TRIA0 differs markedly from those of TRIA 1 and TRIA 2 (see **Figure 1**), primarily due to the presence of a prominent band at 3300 cm^-1^. Furthermore, the broad band at 2668 cm^-1^ is attributed to OH stretching vibration associated with intermolecular hydrogen bonding (Rao, 1963). This band progressively decreased in TRIA 1 and TRIA 2. Notably, the presence of acid groups in different protonation and deprotonation states is confirmed by the bands at 1710 cm^-1^ (C=O stretching motion), 1600 cm^-1^ (asymmetric -COO^-^ stretching motion) and 1240 cm^-1^ (C-O stretching vibration). Additionally, the prominent band detectable at 1030 cm^-1^ is characteristic of C-O groups in primary alcohols (Bellamy, 1975). However, in TRIA 1 and TRIA 2, this band shifts at approximately 70 cm^-1^, indicating an origin from secondary alcohols (Bellamy, 1975). Other bands are at 918 cm^-1^ (-HC=CH- (*cis*-) bending out plain) and at 817 cm^-1^ (*cis* CH out of plane bending).

The chemical analyses revealed higher yield in triacontanol for TRIA1 (plus 17-23%), while TRIA2 had similar triacontanol concentration as the synthetic TRIA0. The total N content was comparable and very low, while the C content was similar between TRIA0 and TRIA1, but lower (minus 34-40%) in TRIA2. The pH was acidic in all extracts, the EC higher for TRIA2 and the density comparable (**Table 1**).

**Table 1:** Chemical characterization of TRIA products tested in this study.

Chemical Characterization	TRIA0	TRIA1	TRIA2
Triacontanol (mg/kg)	6,788	8,000	6,500
Total N (%)	< 0.1	< 0.1	< 0.1
Total C (%)	26.4	28.7	17.4
pH	3.5	3.9	4
EC (dS/m)	0.068	0.05	0.18
Density (kg/L)	1.02	1.03	0.9

Total C, total carbon; total N, total nitrogen; EC, electrical conductivity.

### 3.2 Plant growth and SPAD index

**Figure 2:**
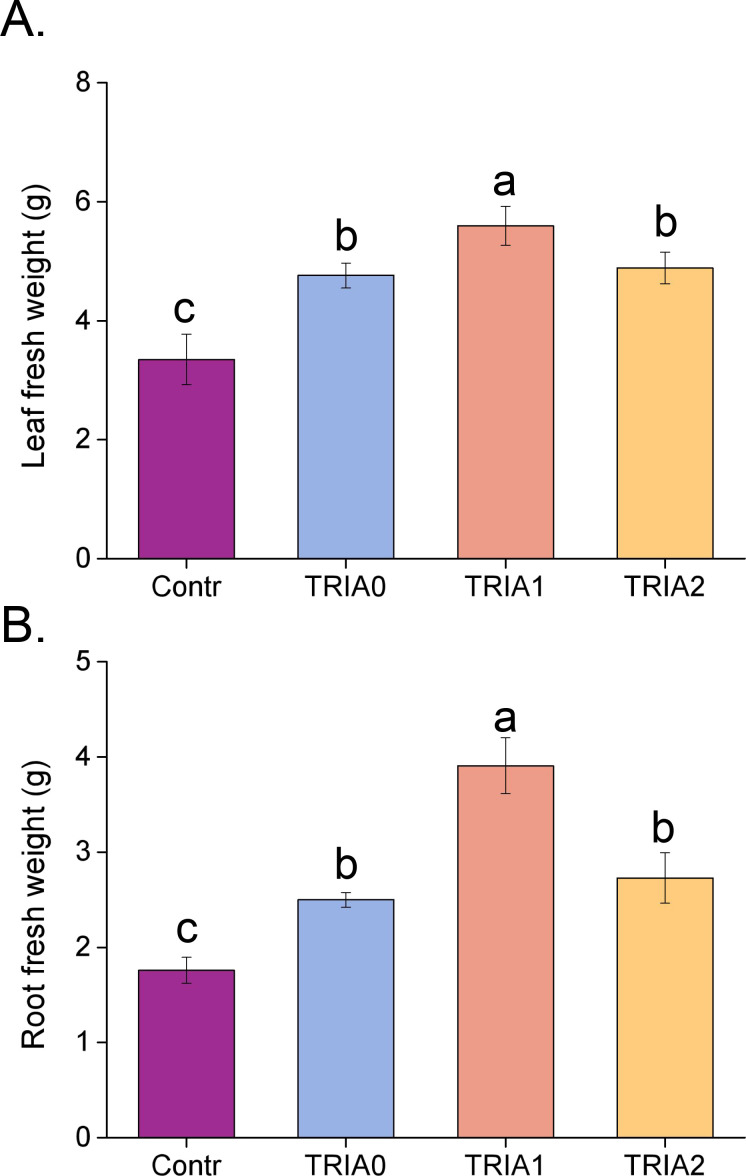
Leaf **(A)** and root **(B)** fresh biomass of rocket plants treated with TRIA extracts (TRIA0, TRIA1, TRIA2) or untreated (control).Data represent the mean of five biological replicates (n=5). Letters above bars indicate significant differences at p <0.05.

TRIA products were applied to rocket plants, and their effects on growth, in terms of fresh and dry biomass production, were evaluated. Results indicated that TRIA0, TRIA1, and TRIA2 caused significant increases in leaf fresh weight by 42%, 67%, and 45%, respectively, compared to the control (**Figure 2A**), with TRIA1 being the most efficient in stimulating growth. A similar trend was observed for the leaf dry weight (**Supplementary Figure 1A**). Regarding roots, a trend in fresh biomass production in response to TRIA treatments was observed that mirrored the pattern found in leaves (**Figure 2B**). The most significant increase in this parameter was observed in plants treated with TRIA1, showing a 122% rise compared to the control. Similarly, increases were also recorded in the roots of plants treated with TRIA0 (42%) and TRIA2 (55%). The dry weight of roots was increased by TRIA treatments, with percent increases of 44%, 53% and 43% for TRIA0, TRIA1 and TRIA2, respectively (**Supplementary Figure 1B**).

**Figure 3:**
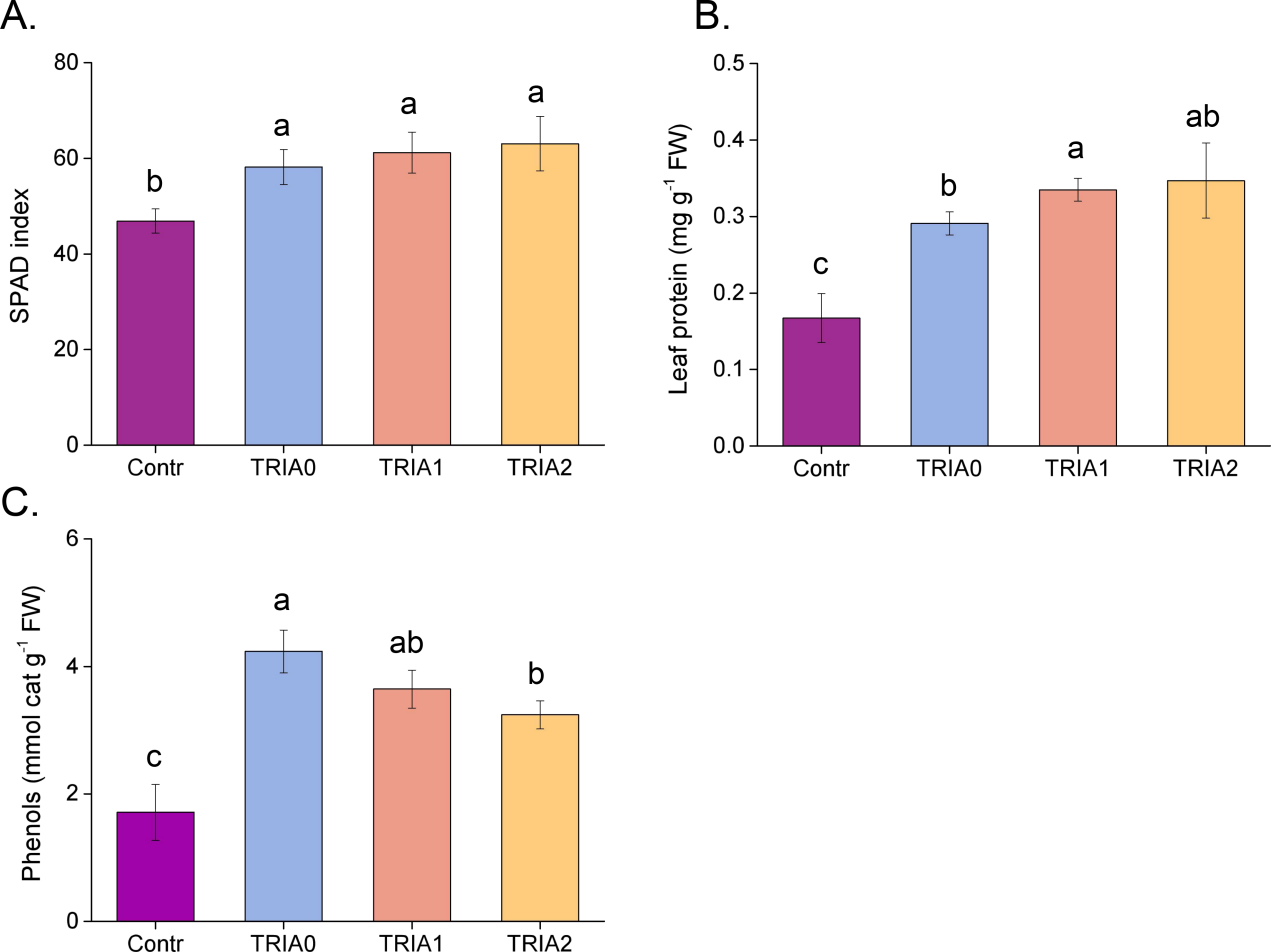
SPAD index **(A)**, leaf protein content **(B)** and total phenol content **(C)** of rocket plants treated with TRIA extracts (TRIA0, TRIA1, TRIA2) or untreated (control). Data represent the mean of three representative biological replicates (n=3). Letters above bars indicate significant differences at p <0.05.

As for the SPAD index, all treatments applied to the rocket plants effectively enhanced photosynthetic efficiency to a similar extent (24-34%), with no significant differences emerging (**Figure 3A**).

### 3.3 Total proteins and phenols quantifications

The protein content in rocket leaves increased in plants treated with the TRIA compounds under study. Specifically, the increases following treatment with TRIA2, TRIA1, and TRIA0 were +108%, +100%, and +74%, respectively (**Figure 3B**). The total phenolic content in rocket leaves also increased across all TRIA-treated plants, with the highest increase observed in plants treated with TRIA0 (+148%) compared to the control (**Figure 3C**).

### 3.4 Elemental analyses

The analysis showed a substantial increase in the concentration of Ca, Fe, P and S in leaves of rocket plants treated with TRIA1 compared to the control (**Table 2**). In plants treated with TRIA0 and TRIA2, no significant variation in leaves nutrient content was observed relative to the control, although values are sometimes comparable to those measured in plants of TRIA1 (e.g., Mg, Fe). In roots, application of products did not lead to significant increases in nutrient content (data not shown).

**Table 2:** Elemental concentration in leaves of rocket plants treated with TRIA extracts (TRIA0, TRIA1, TRIA2) or untreated (control).

Treatment	Ca	K	Mg	P	S	Fe
	(g/kg)					(mg/kg)
Control	16.56 ± 1.23 b	28.95 ± 0.73b	2.87 ± 0.17b	3.39 ± 0.25b	6.04 ± 0.34b	67.03 ± 4.20 c
TRIA0	15.51 ± 1.34 b	28.94 ± 0.59b	2.83 ± 0.25b	3.21 ± 0.29b	5.77 ± 0.48b	70.22 ± 4.13 bc
TRIA1	20.86 ± 1.57 a	33.10 ± 0.65a	3.58 ± 0.18a	4.30 ± 0.17a	7.61 ± 0.56a	84.34 ± 8.10 a
TRIA2	14.69 ± 1.32 b	28.37 ± 0.58b	3.05 ± 0.30ab	3.15 ± 0.29b	6.18 ± 0.42b	77.40 ± 5.01 ab

Data represent the mean of five biological replicates (n=5). Letters along each column indicate significant differences at p <0.05.

### 3.5 Metabolomic analysis

An untargeted metabolomic analysis based on UHPLC/QTOF-MS was conducted to investigate the molecular responses triggered by TRIA products in rocket plants. This comprehensive profiling enabled the putative annotation of over 2106 metabolites, spanning a wide spectrum of biochemical classes involved in both primary and secondary metabolic pathways. The full list of detected compounds, along with composite mass spectra and relative abundances, is provided in the **Supplementary Materials** (**Supplementary Table 2**).

**Figure 4:**
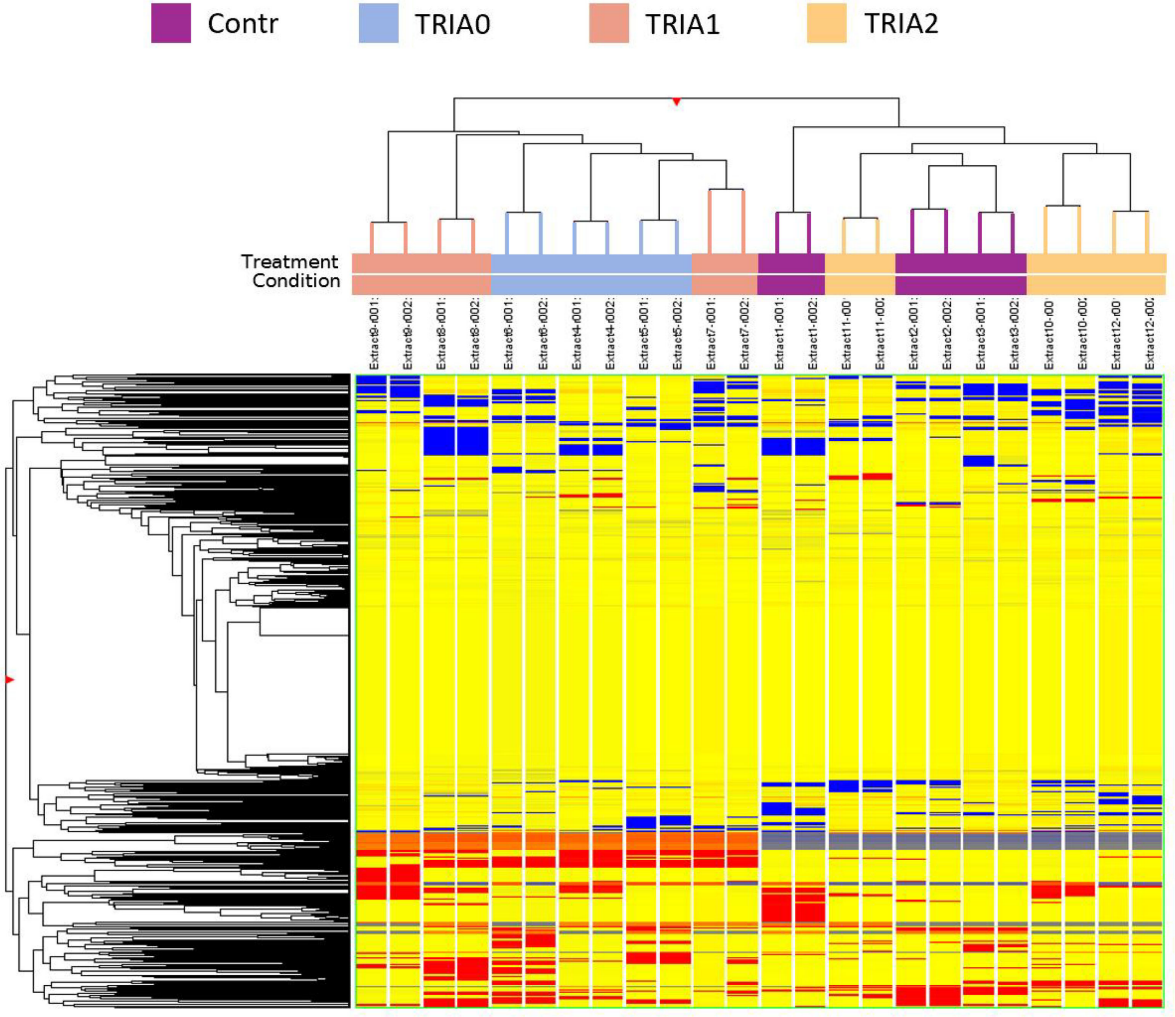
Fold-change heatmap obtained from the unsupervised hierarchical cluster analysis (Euclidean distance; linkage rule: Ward) on UHPLC-ESI/QTOF-MS data of rocket leaf chemical profiles under untreated control, TRIA0, TRIA1 and TRIA2 treatments. Data represent the mean of three representative biological replicates (n=3).

A preliminary unsupervised hierarchical clustering (HCA) bioinformatics analysis was conducted to group plant samples according to their similarities/dissimilarities on their metabolic signatures (**Figure 4**). Two main clusters were identified from the resulting dendrogram, the first of which formed by TRIA0 and TRIA1, while the second one by Control and TRIA2.

**Figure 5:**
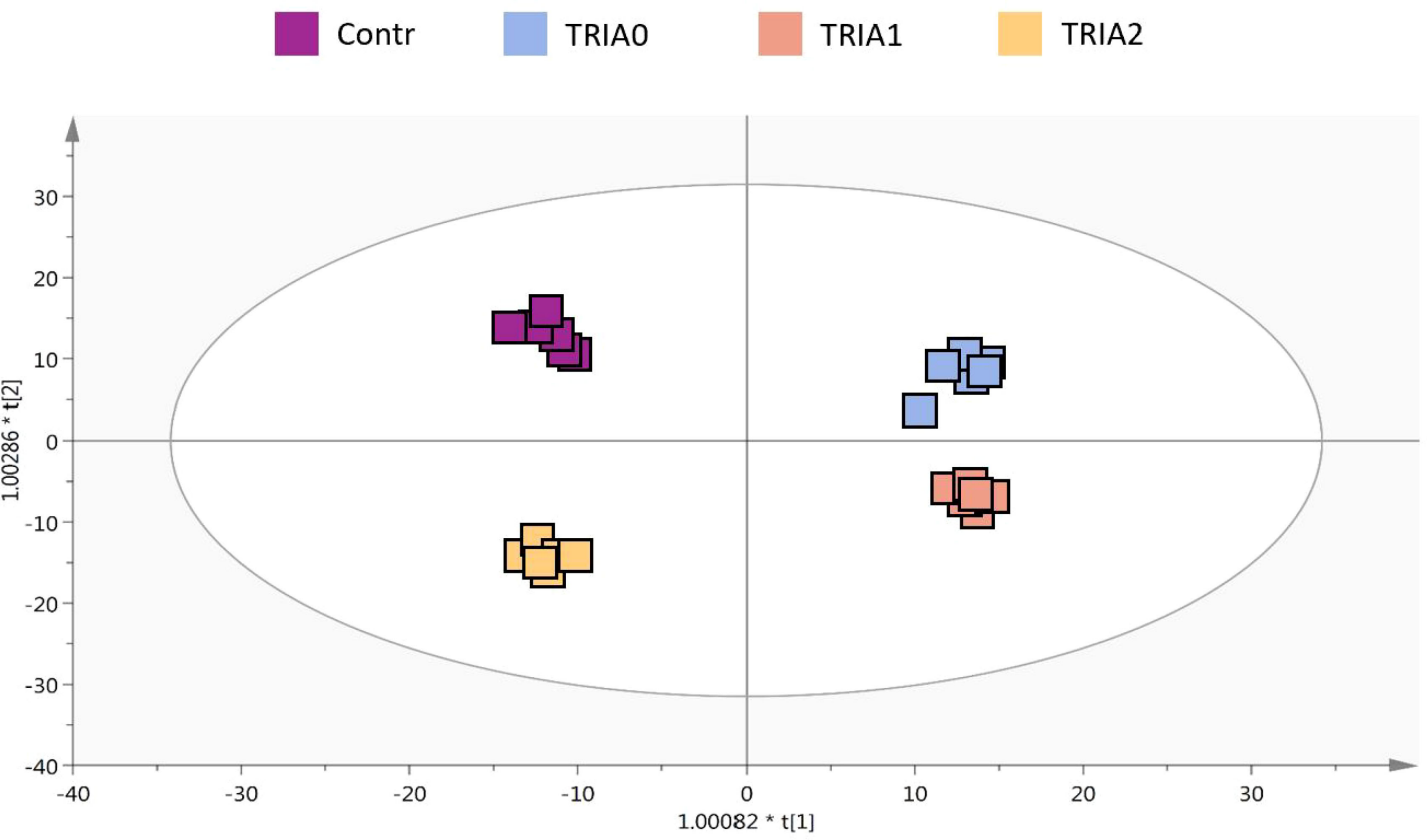
Orthogonal projections to latent structures discriminant analysis (OPLS-DA) score plot for rocket leaf under untreated control, TRIA0, TRIA1 and TRIA2 treatments. Data represent the mean of three representative biological replicates (n=3).

Subsequently, the two-dimensional score plot generated via supervised OPLS-DA multivariate modeling pointed out a robust discrimination among groups based on their metabolomic profiles (**Figure 5**). The first predictive component [t(1)] mainly differentiated groups TRIA0 and TRIA1 (positive side) from TRIA2 and Control (negative side), while the orthogonal component [t(2)] contributed to further separating TRIA0 and Control (upper half) from TRIA1 and TRIA2 (lower half). These findings corroborated the previous HCA results and indicated that the metabolomic profiles differed significantly across the experimental conditions. Model robustness was confirmed by cross-validated ANOVA (CV-ANOVA), which returned a highly significant p-value (1.03 E-05), while the high values of R²Y (0.98) and Q²Y (0.86) reflected excellent model fit and predictive ability. Furthermore, the absence of overfitting was supported by permutation testing performed with more than 100 iterations. To identify the metabolites most relevant for group separation, the VIP scores from the predictive model were used. Compounds with a VIP score ≥ 1.3 were considered highly discriminant, with isoprenoids especially diterpenoids, sesquiterpenes, and carotenoids, alkaloids and phenolics being the most represented classes. The full list of these marker compounds is reported in **Supplementary Table 3**.

**Figure 6:**
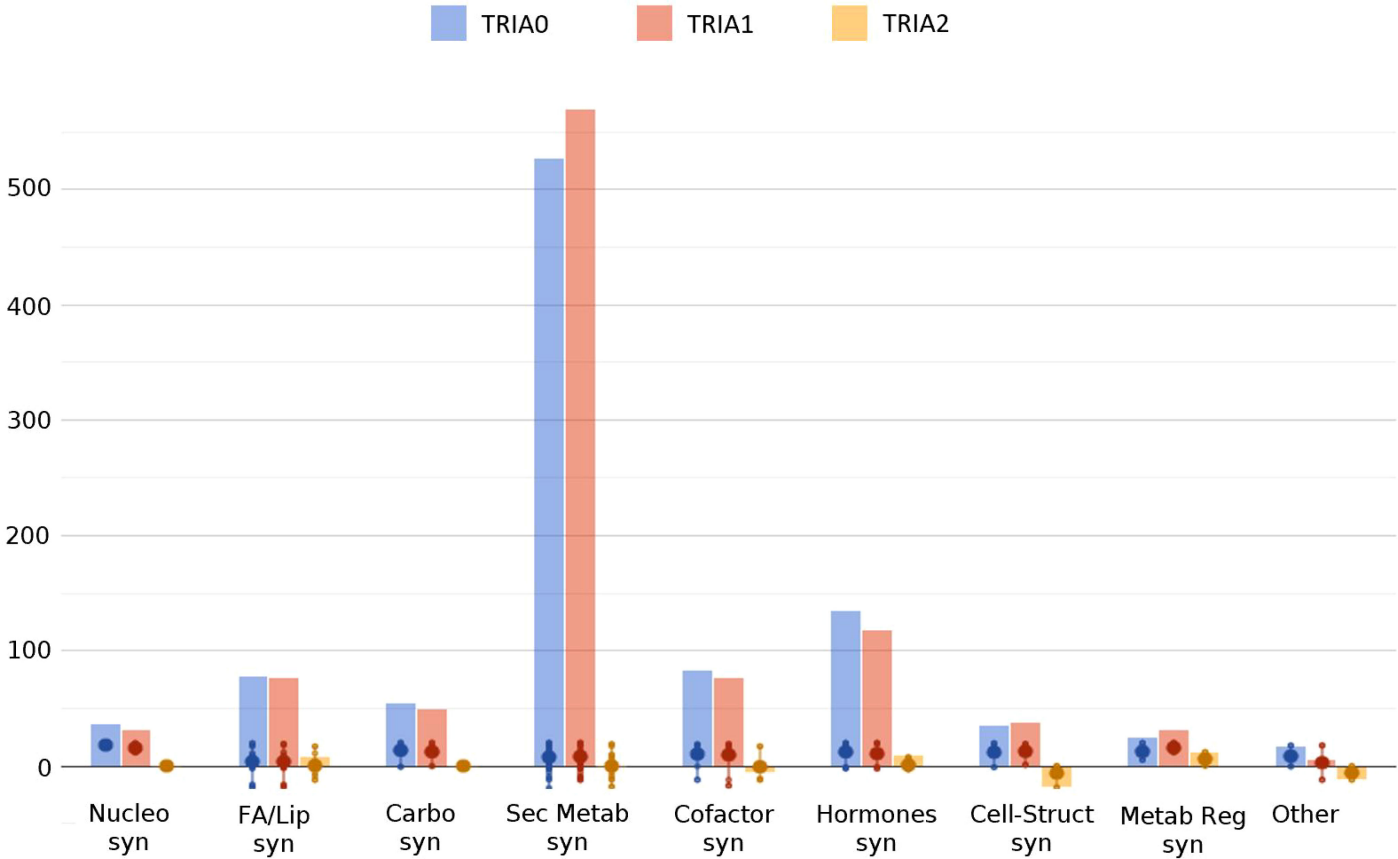
Rocket leaf metabolic processes as affected by TRIA0, TRIA1 and TRIA2 treatments, compared with untreated control (T1). The x-axis represents each set of metabolic subcategories, while the y-axis corresponds to the cumulative log fold change (FC). Data represent the mean of three representative biological replicates (n=3).

Finally, univariate analysis identified 122 significantly modulated metabolites in response to biostimulant treatments compared to the Control (**Supplementary Table 4**). This same subset of differential metabolites was subsequently mapped onto biochemical pathways using the PlantCyc Pathway Tool, as depicted in **Figure 6**. Concerning primary metabolism, lipid and fatty acid biosynthesis was substantially modulated to biostimulant treatments. Specifically, TRIA0 and TRIA1 were associated with higher relative abundances of several lipid components, including 1-palmitoyl-2-linoleoyl-phosphatidylcholine and UDP-α-D-galactose, and various mono- and di- galactosyldiacylglycerols, such as 1-18:1-2-16:0-monogalactosyldiacylglycerol and 1-18:1-2-16:0-digalactosyldiacylglycerol, which may be consistent with changes in membrane-associated lipid metabolism. In contrast, TRIA2 application revealed a weaker response, showing a downregulation of several unsaturated lipids, including 1-α-linolenoyl-2-(3E)-hexadecenoyl-phosphatidylglycerol and 1-18:2-2-18:3-digalactosyldiacylglycerol.

Carbohydrate metabolism was also affected, with TRIA0 and TRIA1 stimulating the accumulation of sugar nucleotides such as UDP-D-apiose and UDP-α-D-galactose. Conversely, TRIA2 was associated with comparatively smaller variations in metabolites belonging to this class.

Among cofactors and vitamins, several compounds like chlorophyll a, 7,8-dihydroneopterin, and thio-molybdenum cofactor showed higher relative abundance in TRIA0- and TRIA1-treated plants, which may suggest modulation of metabolites putatively associated with photosynthetic and redox-related processes. TRIA2, instead, showed lower relative abundance of metabolites putatively annotated such as isochorismate and 3-demethylubiquinol-9, suggesting a metabolic constraint in plastidial cofactor biosynthesis.

Secondary metabolism was the most prominently affected category, particularly in TRIA0- and TRIA1-treated samples, which exhibited a widespread accumulation of specialized compounds. Among them, several metabolites features putatively annotated as alkaloid-related compounds, flavonoids/anthocyanins, and terpenoid derivates showed increased relative abundance in both TRIA1- and TRIA0-treated plants. However, TRIA0 application induced the highest fold changes in several triterpenoid and anthocyanin derivatives. In contrast, TRIA2 induced a mixed response in secondary metabolism, characterized by selective downregulation of flavonoids such as quercetin-3-glucoside, anthraniloyl-O-glucopyranose, and 3-demethylubiquinol-9, which may indicate alterations in metabolites putatively associated with phenylpropanoid-related pathways.

Finally, hormone-related secondary compounds such as brassinosteroids (26-hydroxybrassinolide, brassinolide 22-O-sulfate, castasterone-23-O-glucoside) and jasmonate derivatives ((+)-7-epi-jasmonoyl-CoA) showed higher relative abundance in TRIA0- and TRIA1-treated plants, while TRIA2 again exhibited minimal or inconsistent changes, suggesting a comparatively weaker modulation of metabolites putatively associated with hormonal pathways.

## 4 Discussion

The results of this study highlight the biostimulant effects of TRIA on rocket plants grown in hydroponics and support previous research demonstrating the broad-spectrum efficacy of TRIA in enhancing plant growth and metabolic activity (El-Beltagi et al., 2022; Verma et al., 2023). In addition, they indicate that extraction conditions play a key role in determining the functional properties of TRIA-based products.

TRIA0, TRIA1 and TRIA2 are here considered as process-defined materials, in line with the aim of the current investigation. Within this framework, the different extraction temperatures used to obtain TRIA1 and TRIA2 were associated with distinct biological responses, with TRIA1 containing a higher amount of triacontanol and showing a stronger bioactive effect than TRIA2 at the metabolomic level. Examination of the spectral profiles of TRIA1 and TRIA2, did not reveal clear structural differences, apart from a slightly higher relative intensity of hydroxyl and carbonyl functional groups, features that may be associated with acidic components. In addition, some FT-IR signals could tentatively be associated with a higher relative contribution of cis-configured C=C bonds. However, FT-IR analysis, similarly to other advanced analytical techniques, may not detect subtle differences in composition, crystallinity, particle morphology, surface properties, or the relative abundance of minor co-extracted/degradation products. Since supercritical CO_2_ extraction is strongly influenced by process parameters, including temperature and pressure, the two extraction conditions may have generated materials differing in physicochemical organization or bioavailability, even in the absence of evident spectral variations. Therefore, the greater activity observed for TRIA1 could hypothetically reflect differences in accessibility, dispersion, or interaction with the biological system, rather than substantial structural modifications or the presence of major contaminants. Nevertheless, since no complementary compositional analyses (e.g., GC–MS, LC–MS, or NMR) were performed, no direct conclusions can be drawn regarding possible temperature-induced structural or compositional changes.

It should also be acknowledged that ethanol was used as a solvent during preparation of the treatments. However, the final ethanol concentration in the nutrient solution was very low (0.1%, v/v), well below concentrations commonly associated with phytotoxic or growth-inhibitory effects in plants (≥0.25–1%, v/v; Araujo et al., 2009). Therefore, ethanol is unlikely to have substantially affected the observed physiological or metabolic responses. Nevertheless, the absence of a solvent-only control represents a limitation of the present study, and a minor contribution of the solvent to the observed effects cannot be completely excluded.

Previous studies indicate that TRIA’s ability to enhance plant growth is largely due to its positive effects on photosynthetic efficiency and chlorophyll content (Islam and Mohammad, 2020). In addition, TRIA has been reported to promote lipid biosynthesis and sustain photosynthetic performance under stress conditions. Consistently, our metabolomic data reveal a significant accumulation of membrane-associated sugars and lipid components, essential for chloroplast functionality and light energy utilization (Hölzl and Dörmann, 2019) in TRIA0- and TRIA1- treated plants. In contrast, TRIA2-treated plants exhibited a reduction in unsaturated membrane lipids, suggesting a diminished capacity for lipid remodeling to optimize cellular structures for growth or stress adaptation and overall lower metabolic activity. TRIA has been also reported to promote plant growth by modulating key hormones, including auxins, cytokinins, gibberellins, and abscisic acid (Ahmad et al., 2021), similar to other biostimulants (Ertani et al., 2012; Nardi et al., 2016; Nardi et al., 2018; Ertani et al., 2017; Pizzeghello et al., 2013; Schiavon et al., 2008). Consistent with these reports, our data show that TRIA0 and TRIA1 triggered the accumulation of hormone-related metabolites involved in growth regulation and stress adaptation, particularly those associated with steroidal and jasmonate pathways. In contrast, TRIA2 exhibited limited effects on the accumulation of these hormone-related metabolites, and consequently in hormone-mediated metabolic responses.

The enhanced mineral nutrient content observed in the leaves of TRIA-treated plants, especially in the case of TRIA1, may reflect improved nutrient uptake efficiency. TRIA has been shown to stimulate root growth and upregulate the expression of transporter proteins involved in mineral acquisition (El-Beltagi et al., 2022). Additionally, the increase in protein content in rocket leaves aligns with TRIA’s reported role in enhancing nitrogen assimilation (Islam and Mohammad, 2020). This effect was previously attributed to the TRIA’s ability to stimulate the activity of key enzymes in nitrogen metabolism, thereby facilitating amino acid and protein synthesis (Islam and Mohammad, 2020). In this case, the effects of TRIA1 and TRIA2 on nitrogen content, as indicated by the SPAD index, and on protein accumulation, were greater than those induced by the synthetic TRIA0. This indicated that the efficacy of TRIA2 to promote nitrogen nutrition was not affected by the extraction temperature. A possible explanation is that the biological responses regulated by TRIA are not equally sensitive to the structural or compositional changes induced by high extraction temperatures. As discussed above, the high extraction temperature used to obtain TRIA2 may have partially altered the structural integrity or composition of the extract, thereby reducing its overall biostimulant efficacy. Protein accumulation is generally associated with more direct effects of TRIA on nitrogen assimilation and protein synthesis (Naeem et al, 2011), whereas more complex responses, such as phenolic biosynthesis, broader metabolic reprogramming, and growth stimulation, may depend on a more intact molecular structure and signaling capacity, which could be partially compromised by high temperatures. This may explain why TRIA2 retained the ability to enhance protein content while exhibiting reduced effects on other physiological and metabolic parameters. Finally, secondary metabolism was markedly upregulated in TRIA0 and TRIA1 treatments, as reflected by the accumulation of several classes of bioactive compounds typically linked to plant defense and environmental adaptation. These included phenolic derivatives, nitrogen-containing molecules, and terpenoid-related metabolites, whose combined presence may contribute to improved nutritional quality, as reported in previous literature (Ertani et al., 2015; Ertani et al., 2016; Zhang et al., 2020; Shahrajabian et al., 2022). These findings are consistent with previous research demonstrating that biostimulants, particularly those containing triacontanol, can stimulate the accumulation of secondary metabolites (Zhang and Schmidt, 2000; Ertani et al., 2013). More recently, metabolomic investigations in other Brassicaceae species, such as *Brassica oleracea*, demonstrated that biostimulant treatments can induce extensive reprogramming of secondary metabolic pathways, including metabolites involved in antioxidant defense, stress adaptation, lipid metabolism, and the enhancement of nutraceutical quality (Neamah et al., 2026). By contrast, TRIA2 treatment resulted in a weaker or even repressive modulation of these pathways, especially those related to antioxidant functions and phenylpropanoid metabolism. Thus, while our results confirm that secondary metabolism is a major target of TRIA biostimulant action and that natural TRIA exerts effects on plant metabolism comparable to or greater than the synthetic form, the extraction temperature emerges as a critical factor influencing the molecule’s capacity to regulate secondary metabolic pathways and antioxidant responses.

## 5 Conclusion

The triacontanol materials investigated in this study were differentiated based on their extraction conditions and spectroscopic profiles. The results underscore that TRIA extraction condition may have influenced the functional properties and biological activity of TRIA-based products. In particular, the extraction conditions used to obtain TRIA2 resulted in lower bioactivity of this compound. Although TRIA2 produced the greatest increase in protein content, the differences relative to TRIA0 and TRIA1 were not significant. In terms of growth and SPAD values, TRIA2 elicited responses comparable to TRIA0, but lower than those observed for TRIA1. In addition, TRIA2 induced lower phenolic accumulation and appeared less effective in promoting metabolic changes compared to the other treatments.

Furthermore, this study demonstrates the usefulness of metabolomics in elucidating the molecular effects of biostimulants on plant physiology. By mapping the alterations in metabolite profiles, key biochemical pathways activated in response to TRIA treatment were identified, providing valuable insights for optimizing agricultural practices and enhancing crop resilience.

Although further studies including a larger number of biological replicates would help strengthen the reproducibility of the observed responses across the different analyses performed, the distinct metabolic responses observed among treatments highlight the importance of biostimulant composition and activity in modulating plant physiological and biochemical processes.

Both TRIA0 and TRIA1 exhibited the potential to activate metabolic pathways associated with growth, stress tolerance, and secondary metabolite production, likely due to their higher content of active components. TRIA1, in particular, induced stronger intense growth responses, higher total phenol, protein and element accumulation than the synthetic TRIA0. However, since no targeted compositional analyses were performed, the mechanisms underlying these differences remain hypothetical. As research advances, the applications of triacontanol-based biostimulants may expand further, offering promising opportunities to support crop productivity and resilience under sustainable agricultural systems.

## Data Availability

The original contributions presented in the study are included in the article/**Supplementary Material**. Further inquiries can be directed to the corresponding author.

## Supplementary material

Supplementary Table 1 Principal spectral range and attributions in FT-IR spectra.

Supplementary Table 2 List of metabolites revealed with the untargeted UHPLC-ESI/QTOF-MS analysis on rocket leaves under untreated control, TRIA0, TRIA1 and TRIA2 treatments.

Supplementary Table 3 VIP-selected discriminating compounds from OPLS-DA modeling on rocket leaf metabolic profile, under untreated control (n=3), TRIA0, TRIA1 and TRIA2 treatments.

Supplementary Table 4 List of the 122 compounds selected by ANOVA analysis (p < 0.05, Benjamini correction) and fold change analysis (FC ≥ 1) which were loaded into the PlantCyc pathway tool.

## References

[B1] Ahmad J. Ali A. A. Al-Huqail A. A. Qureshi M. I. (2021). Triacontanol attenuates drought-induced oxidative stress in Brassica juncea L. by regulating lignification genes, calcium metabolism and the antioxidant system. Plant Physiol. Biochem. 166, 985–998. doi: 10.1016/j.plaphy.2021.07.009 34265697

[B2] Ali O. Ramsubhag A. Jayaraman J. (2021). Biostimulant properties of seaweed extracts in plants: Implications towards sustainable crop production. Plants 10, 1–27. doi: 10.3390/plants10030531 33808954 PMC8000310

[B3] Alseekh S. Fernie A. R. (2018). Metabolomics 20 years on: what have we learned and what hurdles remain? Plant J. 94, 933–942. doi: 10.1111/tpj.13950 29734513

[B4] Araujo A. S. Monteiro R. T. R. Abarkeli R. B. (2009). The phytotoxic effect of exogenous ethanol on Euphorbia heterophylla L. Acta Physiol. Plant 31, 821–826. doi: 10.1128/AEM.00491-09 19640725

[B5] Arnaldos T. L. Muñoz R. Ferrer M. A. Calderón A. A. (2001). Changes in phenol content during strawberry (Fragaria x ananassa, cv. Chandler) callus culture. Physiol. Plant 113, 315–322. doi: 10.1034/j.1399-3054.2001.1130303.x 12060275

[B6] Bellamy L. (1975). The Infrared Spectra of Complex Molecules. 1st Edition (Dordrecht: Springer Netherlands), 1–190.

[B7] Bradford M. M. (1976). A rapid and sensitive method for the quantitation of microgram quantities of protein utilizing the principle of protein-dye binding. Anal. Biochem. 72, 248–254. doi: 10.1016/0003-2697(76)90527-3 942051

[B8] Buitrago-Villanueva I. Barbosa-Cornelio R. Coy-Barrera E. (2023). Influence of the culture system and harvest time on the specialized metabolite composition of rocket salad (Eruca sativa) Leaves. Horticulturae 9, 235. doi: 10.3390/horticulturae9020235 30654563

[B5100] Caspi R. Altman T. Billington R. Dreher K. Foerster H. Fulcher C. A. . (2014). The MetaCyc database of metabolic pathways and enzymes and the BioCyc collection of Pathway/Genome Databases. Nucleic Acids Res. 42, D459–D471. doi: 10.1093/nar/gkt1103 24225315 PMC3964957

[B9] Chen X. Yuan H. Chen R. Zhu L. He G. (2003). Biochemical and photochemical changes in response to triacontanol in rice (Oryza sativa L.). Plant Growth Regul. 40, 249–256. doi: 10.1023/A:1025039027270 41886696

[B10] Colautti A. Golinelli F. Iacumin L. Tomasi D. Cantone P. Mian G. (2023). Triacontanol (long-chain alcohol) positively enhances the microbial ecology of berry peel in Vitis vinifera cv. ‘Glera’ yet promotes the must total soluble sugars content. Oeno. One 57, 477–488. doi: 10.20870/oeno-one.2023.57.2.7507

[B11] Drygaś B. Piechowiak T. Balawejder M. Matłok N. Kreczko J. Puchalski C. (2024). The eliciting effect of aqueous extracts from Ascophyllum nodosum algae on the cultivation of arugula (Eruca sativa Mill.) Microgreens. Sustainabil. (Switzerl). 16, 7436. doi: 10.3390/su16177436 30654563

[B12] El-Beltagi H. S. Ismail S. A. Ibrahim N. M. Shehata W. F. Alkhateeb A. A. Ghazzawy H. S. . (2022). Unravelling the effect of triacontanol in combating drought stress by improving growth, productivity, and physiological performance in strawberry plants. Plants 11, 1913. doi: 10.3390/plants11151913 35893617 PMC9330780

[B13] Ertani A. Francioso O. Tugnoli V. Righi V. Nardi S. (2011). Effect of commercial lignosulfonate-humate on Zea mays L. metabolism. J. Agric. Food. Chem. 59, 11940–11948. doi: 10.1021/jf202473e 21999168

[B14] Ertani A. Nardi S. Altissimo A. (2012). Review: Long-term research activity on the biostimulant properties of natural origin compounds. Acta Hortic. 1009, 181–188. doi: 10.17660/actahortic.2013.1009.22

[B15] Ertani A. Pizzeghello D. Francioso O. Tinti A. Nardi S. (2016). Biological activity of vegetal extracts containing phenols on plant metabolism. Molecules 21, 205. doi: 10.3390/molecules21020205 26867189 PMC6273273

[B16] Ertani A. Sambo P. Nicoletto C. Santagata S. Schiavon M. Nardi S. (2015). The use of organic biostimulants in hot pepper plants to help low input sustainable agriculture. Chem. Biol. Technol. Agric. 2, 21. doi: 10.1186/s40538-015-0039-z 38164791

[B17] Ertani A. Schiavon M. Muscolo A. Nardi S. (2013). Alfalfa plant-derived biostimulant stimulate short-term growth of salt stressed Zea mays L. plants. Plant Soil 364, 145–158. doi: 10.1007/s11104-012-1335-z 30311153

[B18] Ertani A. Schiavon M. Nardi S. (2017). Transcriptome-wide identification of differentially expressed genes in Solanum lycopersicon L. In response to an Alfalfa-protein hydrolysate using microarrays. Front. Plant Sci. 8. doi: 10.3389/fpls.2017.01159 28725232 PMC5496959

[B19] Fiehn O. (2002). Metabolomics--the link between genotypes and phenotypes. Plant Mol. Biol. 48, 155–171. doi: 10.1023/a:1013713905833 11860207

[B20] Giordano M. (2021). Effects of plant-based biostimulants, used alone or in combination, on yield and quality of rocket plants. Biol. Life. Sci. Forum 2021, 1–15. doi: 10.3390/horticulturae7050107

[B21] Hölzl G. Dörmann P. (2019). Chloroplast lipids and their biosynthesis. Annu. Rev. Plant Biol. 70, 51–81. doi: 10.1146/annurev-arplant-050718-100202 30786236

[B22] Islam S. Mohammad F. (2020). Triacontanol as a dynamic growth regulator for plants under diverse environmental conditions. Physiol. Mol. Biol. Plants 26, 871–883. doi: 10.1007/s12298-020-00815-0 32377038 PMC7196594

[B23] Lee S. W. Nugroho A. B. D. Nugroho D. Kim D. H. (2024). Correlation analysis of glucosinolate profiles and GSL biosynthetic genes in radishes (Raphanus sativus L.). Hortic. Environ. Biotechnol. 65, 157–167. doi: 10.1007/s13580-023-00555-6 30311153

[B24] Lucini L. Rouphael Y. Cardarelli M. Canaguier R. Kumar P. Colla G. (2015). The effect of a plant-derived biostimulant on metabolic profiling and crop performance of lettuce grown under saline conditions. Sci. Hortic. 182, 124–133. doi: 10.1016/j.scienta.2014.11.022 38826717

[B26] Manai M. Fiorillo A. Matuozzo M. Li M. D’Ambrosio C. Franco L. . (2024). Phenotypical and biochemical characterization of tomato plants treated with triacontanol. Sci. Rep. 14, 62398. doi: 10.1038/s41598-024-62398-0 38802434 PMC11130248

[B27] Meenakshi S. Gnanambigai D. M. Mozhi S. T. Arumugam M. Balasubramanian T. (2009). Total flavonoid and *in vitro* antioxidant activity of two seaweeds of Rameshwaram coast. Global J. Pharmacol. 3, 59–62.

[B28] Mollo L. Petrini A. Norici A. Ferrante A. Cocetta G. (2025). Enhanced growth and photosynthetic efficiency in wild rocket (Diplotaxis tenuifolia L.) following multi-species microalgal biostimulant application. Plant Sci. 359, 112643. doi: 10.1016/j.plantsci.2025.112643 40618902

[B5000] Murashige T. Skoog F. (1962). A revised medium for rapid growth and bioassays with tobacco tissue cultures. Physiol. Plant. 15, 473–497. doi: 10.1111/j.1399-3054.1962.tb08052.x

[B29] Naeem M. Khan M. M. A. Moinuddin Idrees M. Aftab T. (2011). Triacontanol-mediated regulation of growth and other physiological attributes, active constituents and yield of Mentha arvensis L. Plant Growth Regul. 65, 195–206. doi: 10.1007/s10725-011-9588-8 30311153

[B30] Nanda S. Kumar G. Hussain S. (2022). Utilization of seaweed-based biostimulants in improving plant and soil health: Current updates and future prospective. Int. J. Environ. Sci. Technol. 19, 12839–12852. doi: 10.1007/s13762-021-03568-9 30311153

[B31] Nardi S. Pizzeghello D. Ertani A. (2018). Hormone-like activity of the soil organic matter. Appl. Soil Ecol. 123, 517–520. doi: 10.1016/j.apsoil.2017.04.020 38826717

[B32] Nardi S. Pizzeghello D. Schiavon M. Ertani A. (2016). Plant biostimulants: Physiological responses induced by protein hydrolyzed-based products and humic substances in plant metabolism. Sci. Agric. 73, 18–23. doi: 10.1590/0103-9016-2015-0006 41099703

[B34] Neamah W. H. Al-Sudani Z. A. Hasan F. A. Al-Hashemi F. H. Ahlawat Y. K. Sharma P. . (2026). Biostimulants combination enhance the metabolites content of brassica Oleracea var. Botrytis curds cultivated in the desert region of Basrah Governorate. BMC Plant Biol. 26, 653. doi: 10.1186/s12870-026-08237-y 41781856 PMC13067605

[B35] Parrey Z. A. Islam S. Shah S. H. Mohammad F. (2023). Agronomical strategies to improve growth, physio-biochemistry, yield and quality attributes of mint plants under the varied environmental conditions: a Review. J. Soil Sci. Plant Nutr. 23, 1489–1514. doi: 10.1007/s42729-023-01194-7 30311153

[B36] Pizzeghello D. Francioso O. Ertani A. Muscolo A. Nardi S. (2013). Isopentenyladenosine and cytokinin-like activity of different humic substances. J. Geochem. Explor. 129, 70–75. doi: 10.1016/j.gexplo.2012.10.007 38826717

[B37] Rao (1963). Chemical Applications of Infrared Spectroscopy (New York and London: Academic Press).

[B4900] Ries S. K. Savithiry S. D. Wert V. F. Widders I. E. (1993). Rapid induction of ion pulses in tomato, cucumber, and maize plants following a foliar application of L(+)-adenosine. Plant Physiol. 101, 49–55. doi: 10.1104/pp.101.1.49 12231664 PMC158646

[B38] Sarwar M. Anjum S. Ali Q. Alam M. W. Haider M. S. Mehboob W. (2021). Triacontanol modulates salt stress tolerance in cucumber by altering the physiological and biochemical status of plant cells. Sci. Rep. 11, 23063. doi: 10.1038/s41598-021-04174-y 34969963 PMC8718522

[B39] Schiavon M. Ertani A. Nardi S. (2008). Effects of an alfalfa protein hydrolysate on the gene expression and activity of enzymes of the tricarboxylic acid (TCA) cycle and nitrogen metabolism in Zea mays L. J. Agric. Food. Chem. 56, 11800–11808. doi: 10.1021/jf802362g 19053364

[B40] Schiavon M. Nardi S. Pilon-Smits E. A. H. Dall’Acqua S. (2022). Foliar selenium fertilization alters the content of dietary phytochemicals in two rocket species. Front. Plant Sci. 13. doi: 10.3389/fpls.2022.987935 36119625 PMC9470978

[B41] Shahrajabian M. H. Cheng Q. Sun W. (2022). The effects of amino acids, phenols and protein hydrolysates as biostimulants on sustainable crop production and alleviated stress. Recent Pat. Biotechnol. 16, 319–328. doi: 10.2174/1872208316666220412133749 35418295

[B42] Shukla P. S. Mantin E. G. Adil M. Bajpai S. Critchley A. T. Prithiviraj B. (2019). Ascophyllum nodosum-based biostimulants: Sustainable applications in agriculture for the stimulation of plant growth, stress tolerance, and disease management. Front. Plant Sci. 10. doi: 10.3389/fpls.2019.00655 31191576 PMC6548832

[B43] Sumner L. W. Amberg A. Barrett D. Beale M. H. Beger R. Daykin C. A. . (2007). Proposed minimum reporting standards for chemical analysis. Metabolomics 3, 211–221. doi: 10.1007/s11306-007-0082-2 24039616 PMC3772505

[B44] Verma T. Bhardwaj S. Raza A. Djalovic I. Prasad P. V. Kapoor D. (2023). Mitigation of salt stress in Indian mustard (Brassica juncea L.) by the application of triacontanol and hydrogen sulfide. Plant Signal. Behav. 18, 2189371. doi: 10.1080/15592324.2023.2189371 36934336 PMC10026909

[B45] Verma T. Bhardwaj S. Singh J. Kapoor D. Prasad R. (2022). Triacontanol as a versatile plant growth regulator in overcoming negative effects of salt stress. J. Agric. Food Res. 10, 100351. doi: 10.1016/j.jafr.2022.100351 38826717

[B46] Weremczuk-Jeżyna I. Hnatuszko-Konka K. Lebelt L. Piotrowska D. G. Grzegorczyk-Karolak I. (2022). The effect of the stress-signalling mediator triacontanol on biochemical and physiological modifications in Dracocephalum forrestii culture. Int. J. Mol. Sci. 23, 15147. doi: 10.3390/ijms232315147 36499476 PMC9735700

[B47] Yang T. Samarakoon U. Altland J. Ling P. (2021). Photosynthesis, biomass production, nutritional quality, and flavor-related phytochemical properties of hydroponic-grown arugula (Eruca sativa Mill.) ‘standard’ under different electrical conductivities of nutrient solution. Agronomy 11, 1340. doi: 10.3390/agronomy11071340 30654563

[B49] Zhang X. Schmidt R. E. (2000). Hormone-containing products’ impact on antioxidant status of tall fescue and creeping bentgrass subjected to drought. Crop Sci. 40, 1344–1349. doi: 10.2135/cropsci2000.4051344x

[B48] Zhang B. Zhang Y. Li H. Deng Z. Tsao R. (2020). A review on insoluble-bound phenolics in plant-based food matrix and their contribution to human health with future perspectives. Trends Food Sci. Technol. 105, 347–362. doi: 10.1016/j.tifs.2020.09.029 38826717

