# Supplementary material for: Biostimulant potential of triacontanol extracted from a legume-derived hydrolysate in Eruca sativa (L.)
Source: Front Plant Sci. 2026 Jun 30;17:1847175. doi: 10.3389/fpls.2026.1847175 (PMC13364671; doi:10.3389/fpls.2026.1847175)
Supplement: Supplementary Table 1 — Principal spectral range and attributions in FT-IR spectra. [file SupplementaryFile1.docx]

Table S1. Principal spectral range and attributions.

| **Spectral range cm^-^**^1^ | **Band assignments** |
| --- | --- |
| 3650-3200 | O–H, H-bonded OH |
| 2960-2852 | Aliphatic CH |
| 1760-1650 | R–C=O in esters; C=O in acids |
| 1650-1500 | C=O in acids; COO^–^_;_ C=C aliphatic chains |
| 1457-1350 | Aliphatic CH; COO^–^ and O–H |
| 1300-1200 | C–O in acid and esters; C-OH |
| 1200-900 | C-O in primary alcohols |
