# Supplementary figures and images for: Biostimulant potential of triacontanol extracted from a legume-derived hydrolysate in Eruca sativa (L.)
Source: Front Plant Sci. 2026 Jun 30;17:1847175. doi: 10.3389/fpls.2026.1847175 (PMC13364671; doi:10.3389/fpls.2026.1847175)

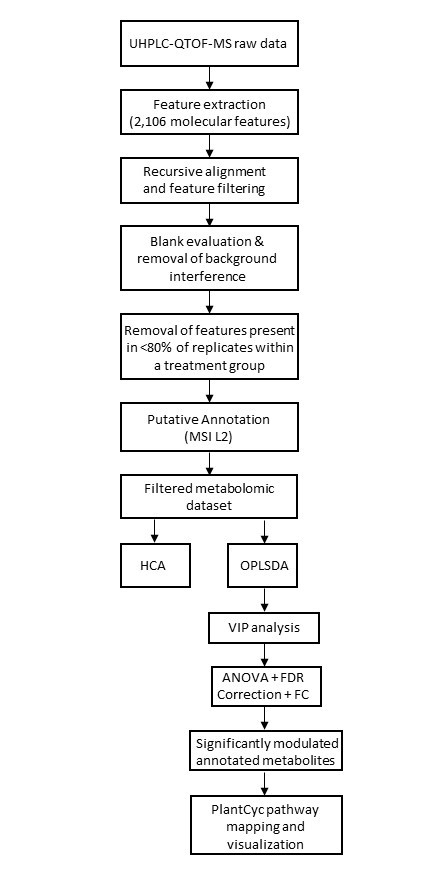

Supplement: Supplementary file 3 [file Image1.jpeg]
